# Multisensor Data Fusion in IoT Environments in Dempster–Shafer Theory Setting: An Improved Evidence Distance-Based Approach

**DOI:** 10.3390/s23115141

**Published:** 2023-05-28

**Authors:** Nour El Imane Hamda, Allel Hadjali, Mohand Lagha

**Affiliations:** 1 ASL, Aeronautics and Spatial Studies Institute, Blida 1 University, Blida 09000, Algerialaghamohand@univ-blida.dz (M.L.); 2 LIAS, National Engineering School for Mechanics and Aerotechnics, 86961 Futuroscope Chasseneuil, France

**Keywords:** multisensor data fusion, IoT, Dempster–Shafer theory, uncertainty, evidence distance, belief entropy

## Abstract

In IoT environments, voluminous amounts of data are produced every single second. Due to multiple factors, these data are prone to various imperfections, they could be uncertain, conflicting, or even incorrect leading to wrong decisions. Multisensor data fusion has proved to be powerful for managing data coming from heterogeneous sources and moving towards effective decision-making. Dempster–Shafer (D–S) theory is a robust and flexible mathematical tool for modeling and merging uncertain, imprecise, and incomplete data, and is widely used in multisensor data fusion applications such as decision-making, fault diagnosis, pattern recognition, etc. However, the combination of contradictory data has always been challenging in D–S theory, unreasonable results may arise when dealing with highly conflicting sources. In this paper, an improved evidence combination approach is proposed to represent and manage both conflict and uncertainty in IoT environments in order to improve decision-making accuracy. It mainly relies on an improved evidence distance based on Hellinger distance and Deng entropy. To demonstrate the effectiveness of the proposed method, a benchmark example for target recognition and two real application cases in fault diagnosis and IoT decision-making have been provided. Fusion results were compared with several similar methods, and simulation analyses have shown the superiority of the proposed method in terms of conflict management, convergence speed, fusion results reliability, and decision accuracy. In fact, our approach achieved remarkable accuracy rates of 99.32% in target recognition example, 96.14% in fault diagnosis problem, and 99.54% in IoT decision-making application.

## 1. Introduction

The IoT environment consists of tiny sensor-enabled connected devices or things able of sensing, communicating, computing, and reasoning. Data generated in IoT environments are voluminous, with diverse representations, different qualities, and different reliability levels. Additionally, due to multiple factors such as environmental noise, sensor defects, or calibration errors, these data are prone to various imperfections. Then, they could be noisy, uncertain, conflicting, or even erroneous, which may lead to wrong decisions if those features are not taken into account properly.

Multisensor data fusion has been proven to be a very powerful technique to manage data coming from heterogeneous sources and to move towards effective decision-making. It aims to combine data gathered from various sensors in the best possible manner to get more precise, accurate, and consistent information. Multisensor data fusion technique can be performed at various levels, depending on the representation of data to be merged and according to the stage at which the combining operation takes place. One can distinguish low, intermediate, and high-level fusion [1]. Several mathematical methods are used for the data fusion process; they are mainly classified into three categories: probability-based, artificial intelligence-based, and evidence-based techniques [2]. Evidence theory, also known as the theory of belief functions or Dempster–Shafer theory (or D–S theory in short), is a robust and flexible mathematical tool for modeling and merging uncertain, imprecise, and incomplete data. The theory was first introduced by Dempster in 1967 [3] as a generalization of Bayesian inference, and then further extended by his student Shafer in 1976 [4] into a general framework of uncertain reasoning.

D–S theory has been extensively applied in various multisensor data fusion applications such as decision-making [5,6,7], fault diagnosis [8,9,10,11], target recognition [12,13,14], etc., owing to its flexibility and effectiveness in handling uncertainty problems and its ability in merging heterogeneous data obtained from multiple sensors without prior knowledge using Dempster’s combination rule. However, the application of D–S evidence theory has its own limitations when dealing with highly conflicting multisensor data; Dempster’s combination rule can lead to counter-intuitive results as illustrated by the conflictive example known as the Zadeh paradox [15]. To conquer this flaw and obtain reasonable combined results, several alternatives have been proposed in the literature; they are mainly divided into two major categories: (i) the modification of the classical Dempster’s combination rule and (ii) the revision of the original evidence model before combination.

For the first category, scholars believe that the unreasonable results come from the direct normalization of the conflicting evidence, so they have proposed new combination rules [16,17,18,19,20]. These rules manage to solve the problem to some extent, but they lose both commutativity and associativity properties satisfied by the classical Dempster’s combination rule. According to the researchers of the 2nd category, the solution lies in reducing the conflicting evidence impact on the final fusion result, so they proposed pre-processing the bodies of evidence before combining them [21,22,23,24,25,26,27,28,29,30,31,32,33,34,35,36,37,38,39,40,41,42,43,44,45,46,47,48,49,50,51,52,53,54,55,56,57,58,59,60,61,62,63,64,65,66,67,68,69,70,71,72,73,74,75,76,77,78,79,80,81,82,83,84,85,86,87,88,89,90,91]. To this end, different distances between bodies of evidence and various entropies (to estimate the uncertainty of the bodies of evidence) are used. For instance, Murphy [21] suggested simply averaging the mass functions and then combining them using the classical Dempster’s combination rule, but it seems unreasonable to assign equal weights to all the bodies of evidence without taking the correlation between them into consideration. Yong et al. [22] proposed a weighted average combination rule based on evidence distance (i.e., Jousselme distance) to measure the conflict degree between the bodies of evidence. Zhang [25] proposed an improved combining method based on the degree of support between the bodies of evidence using the cosine theorem. In our recent work [86], we combined the Jousselme distance and the cosine value to determine the conflict degree between the bodies of evidence, and we adopted Deng entropy to measure the uncertainty degree of each body of evidence; we used both conflict and uncertainty measurements to construct weighting factors to modify the original mass functions. Tang et al. [88] proposed a new approach to pre-process evidence by introducing a reliability coefficient based on the betting commitment evidence distance and the single-factor belief function. The betting commitment is constructed on the basis of the pignistic probability function to measure the dissimilarity between two BPAs, while the single factor belief function is a single subset used to evenly distribute the probability to each subset of the power set. In [89], the evidence model was revised using a novel correction coefficient based on a stochastic approach for the link-structure analysis (SALSA) algorithm combined with the Lance–Williams distance to better measure the degree of support for each piece of evidence. Ma et al. [90] adopted the trusted discount method to alleviate the shortcoming in D–S theory, incorporating Jousselme distance for conflict measurement, and Wasserstain distance for uncertainty measurement, and they proposed a new adaptive weights’ allocation method. Huwa and Jing [91] introduced an improved belief Hellinger divergence that considers both belief and plausibility functions to measure the discrepancy between the pieces of evidence and used belief entropy to measure the uncertainty degree. While several methods and alternatives have been proposed in the literature, effectively addressing the conflict between evidence continues to be an open issue that requires further improvement.

In this paper, an improved evidence combination method for multisensor data fusion in IoT environments is proposed to overcome the Dempster–Shafer theory flaw and fuse highly conflicting evidence without generating counter-intuitive results. It is based on a newly defined evidence distance and belief entropy. The method aims to evaluate the importance of each source in the fusion process by assigning evidence weights. The main idea is to weight the reliable sources most heavily and assign lower weights to the less reliable ones. This strategy intends to reduce the conflicting impact of less reliable sources on the final combination result, thereby enhancing decision-making accuracy.

The main contributions of this study are summarized as follows:

- We first propose an improved evidence distance based on Hellinger distance, which can effectively quantify the conflict degree between the pieces of evidence.
- The improved evidence distance considers the dependencies between the bodies of evidence through a Jaccard matrix, it satisfies true metric properties (non-negativity, symmetry, positive definiteness, and trigonometric inequality), and it allows a better conflict measurement.
- We develop a novel multisensor data fusion strategy based on the improved evidence distance and Deng entropy for conflict and uncertainty measurements, respectively. Reward and penalty functions are designed to assign the weights accordingly. We reward the reliable pieces of evidence by weighting them more heavily, making their role more important in the final fusion result and, conversely, for the less reliable ones.
- Finally, we apply the proposed approach to a benchmark example for target recognition, a fault diagnosis problem, and an IoT decision-making application to demonstrate its effectiveness compared to other similar existing methods. The results have shown the superiority and efficiency of the proposed method in terms of conflict management, convergence speed, fusion results reliability, and decision accuracy.

The remainder of this work is organized as follows: Section 2 introduces some relevant basic theoretical foundations, including Dempster–Shafer theory, Zadeh paradox, Hellinger distance, and belief entropy, and a review of the main related works is outlined in Section 3. The proposed improved evidence distance is presented in Section 4, followed by the proposed evidence combination method in Section 5. In Section 6, the simulation of fusion results and comparisons with similar existing methods are presented and discussed. Conclusions and future research directions are provided in Section 7.

## 2. Theoretical Foundations

In this section, some relevant preliminaries are introduced. First, we provide in Table 1 the symbols with their meanings used in the rest of the paper.

### 2.1. Dempster–Shafer Theory

Dempster–Shafer theory (D–S), also known as evidence theory or the theory of belief functions, is an effective mathematical method for reasoning under uncertainty; it was first initiated by A.P. Dempster in 1967 [3] as a generalization of Bayesian inference, and then developed by his student Shafer in 1976 [4] into a general framework of uncertain reasoning, by introducing the concept of “trust function”. Unlike probability theory, D–S theory doesn’t only allow for the allocation of a probability mass to mutually exclusive singletons, but also to sets or intervals, and it doesn’t require prior knowledge to combine the pieces of evidence.

#### Basic Definitions

**Definition** **1.** *Frame of discernment:*

*Denoted by Ω, it is a finite, nonempty set of mutually exclusive and exhaustive hypotheses,* $A_{i}$*. It is expressed as follows:*

(1) $$\Omega =\left\{A_{1},\;A_{2},\ldots ,A_{n}\right\},$$

*The power set of Ω, denoted by* $2^{\Omega }$ *, is a set of all the possible combinations of all the elements in Ω. For all A ⊆ Ω, it is defined as follows:*

(2) $$2^{\Omega }=\left\{\emptyset ,\;\left\{A_{1}\right\}\;,\ldots \;\left\{A_{n}\right\},\;\left\{A_{1}\cup A_{2}\right\},\ldots ,\;\Omega \right\},$$

**Definition** **2.** *Basic Probability Assignment, BPA (or Mass function)*

*BPA represents how strongly the evidence supports a hypothesis by assigning a probability to the different subsets. In a discernment frame, the mass function of a subset symbolized by m is defined as follows:*

(3) $$m\;:\;2^{\Omega }\to \;\left[0,1\right],$$

*satisfying the following conditions:*

(4) $$\left\{\begin{array}{c} m\left(\emptyset \right)=0\\ \underset{A\subseteq \Omega }{\sum }m\left(A\right)=1 \end{array}\right.$$

∀*A* ⊆ *Ω, if m(A) > 0, A is called a focal element of evidence.*

**Definition** **3.** *Uncertainty representation*

*Based on the definition of BPAs, belief function (Bel) and plausibility function (Pl), which represent, respectively, the lower and upper bounds of the uncertainty interval, are defined as follows:*

(5) $$Bel\left(A_{i}\right)=\underset{A_{j}\;\subset \;A_{i}}{\sum }m\left(A_{j}\right)$$

(6) $$Pl\left(A_{i}\right)=\underset{A_{j}\cap A_{i}=\emptyset }{\sum }m\left(A_{j}\right)$$

*where the belief function represents the total belief in hypothesis A to be true, while the plausibility function refers to the possible belief in the Hypothesis A.*

**Definition** **4.** *Evidence combination rule*

*In evidence theory, the two BPAs* $m_{1}$ *and* $m_{2}$ *under the same frame of discernment, separately obtained from two independent sources, can be combined using Dempster’s combination rule, which provides a method to compute the orthogonal sum denoted by* $m_{1}⊕\;m_{2}\;$ *as follows:*

(7) $$m_{1}⊕m_{2}\left(A\right)=\left\{\begin{array}{c} 0\;\;\;if\;A=\emptyset \\ \frac{1}{1-K}\underset{A_{i}\cap A_{j}=A}{\sum }m_{1}\left(A_{i}\right)m_{2}\left(A_{j}\right)\;\;if\;A\;\neq \;\emptyset \end{array}\right.\;$$

(8) $$K=\underset{A_{i}\cap A_{j}=\emptyset }{\sum }m_{1}\left(A_{i}\right)m_{2}\left(A_{j}\right)\;$$

*where K is the conflict coefficient used to measure the conflict degree between two bodies of evidence. The case K = 0 means that the sources are consistent and in perfect agreement, while K = 1 implies that the sources are in total conflict.*

*Dempster’s combination rule satisfies both commutativity and associativity properties as follows:*

(9) $$\left\{\begin{array}{c} m_{1}⊕m_{2}=m_{2}⊕m_{1}\\ \left(m_{1}⊕m_{2}\right)⊕m_{3}=m_{1}⊕\;\left(m_{2}⊕m_{3}\right) \end{array}\right.$$

*thus, it can be extended to the combination of N bodies of evidence.*

*It should be noted that Dempster’s combination rule is efficient only when the pieces of evidence are not in conflict, unreasonable results will be generated when the sources are contradictory, as highlighted in Zadeh’s counter-example* [15].

### 2.2. Zadeh Paradox

Suppose a diagnosis of a patient from two doctors with three possible diseases, i.e., meningitis (*M*), brain tumor (*BT*), or concussion (*C*). The frame of discernment is then defined as follows: Ω = {*M*, *BT*, *C*}. The two doctors express their opinions, and the results are interpreted as mass functions as follows:

$$m_{1}\left(\left\{M\right\}\right)=0.99;\;\;\;\;\;\;\;m_{1}\left(\left\{BT\right\}\right)=0.01;$$

$$m_{2}\left(\left\{C\right\}\right)=0.99;\;\;\;\;\;\;\;m_{2}\left(\left\{BT\right\}\right)=0.01\;$$

By combining these two pieces of evidence with Dempster’s combination rule, one can obtain the following:

$$m\left(\left\{M\right\}\right)=0;\;m\left(\left\{\mathrm{BT}\right\}\right)=1;\;m\left(\left\{C\right\}\right)=0$$

It’s clear that the combined result is counter-intuitive, it shows that the patient suffers from a brain tumor, the possibility of which both of the doctors claim a very low degree of belief. In contrast, the possibility of meningitis and concussion, which were strongly supported by one of the bodies of evidence, are completely denied after using Dempster’s combination rule. This proves the ineffectiveness of this combination rule in such situations.

### 2.3. Hellinger Distance between Bodies of Evidence

Hellinger distance is a complete distance metric defined in the probability distribution space. It is considered as the probabilistic analog of the Euclidean distance. It was expressed in terms of the Hellinger integral initiated by Hellinger in 1909. Hellinger distance is very stable and reliable, and it is widely used to measure the dissimilarity of two probability distributions, and it can be applied to evidence theory to measure the dissimilarity between two pieces of evidence.

In a finite complete frame of discernment, the Hellinger distance between two bodies of evidence is defined as follows [61]:

(10) $$d_{H}\left(m_{1},m_{2}\right)=\frac{1}{\sqrt{2}}\overset{n}{\underset{i=1}{\sum }}\|\sqrt{m_{1}\left(A_{i}\right)}-\sqrt{m_{2}\left(A_{i}\right)}{\|}_{2}$$

Hellinger distance satisfies the following properties:

1. Non-negativity: $0\leq \;d_{H}\left(m_{1},m_{2}\right)\leq 1$
2. Symmetry: $d_{H}\left(m_{1},m_{2}\right)=d_{H}\left(m_{2},m_{1}\right)$
3. Triangle inequality: $d_{H}\left(m_{1},m_{2}\right)+d_{H}\left(m_{2},m_{1}\right)\geq d_{H}\left(m_{1},m_{3}\right)\;$
4. Positive definiteness: $d_{H}\left(m_{1},m_{2}\right)=0$, if and only if $m_{1}=m_{2}$

Hellinger distance is an effective tool to quantify conflict degrees between pieces of evidence, The smaller the distance is, the more similar and less conflicting the bodies of evidence.

### 2.4. Deng Entropy

The concept of entropy was first introduced in physics to characterize the disorder and chaos degree of a molecular state in thermodynamics [92]. In information theory, Shannon entropy [93] was proposed to measure the uncertainty of information under the framework of probability theory. Deng entropy is a belief entropy, proposed by Deng [27] as a generalization of Shannon entropy, defined under the Dempster–Shafer framework; it is a very efficient tool to quantify the uncertainty degree of the evidence. It takes into consideration the BPA of a hypothesis and the cardinality of the element of the BPA; it is given by the following:

(11) $$E_{d}=-\sum_{i}m\left(A_{i}\right)log\frac{m\left(A_{i}\right)}{2^{\left|A_{i}\right|}-1\;}$$

where $A_{i}$ represents a hypothesis of a belief function m and |$A_{i}$| is the cardinality of the set $A_{i}$.

Deng entropy definitely degenerates to Shannon entropy when the mass function is only allocated to singletons (single elements), as follows:

(12) $$\;E_{s}=-\sum_{i}m\left(A_{i}\right)log\;m\left(A_{i}\right)$$

## 3. Related Works

As mentioned in the Introduction, one of the major problems with Dempster’s combination rule is the fact that it leads to counter-intuitive results when fusing highly conflicting evidence. To solve such a problem, two major methodologies are popular. One is to modify the combined rule, and the other is to preprocess the bodies of evidence.

For the first methodology, scholars believe that the unreasonable results come from the direct normalization of the conflicting evidence, so they have proposed new combination rules. According to Yager [16], conflicting data don’t provide useful information, so he proposed to transfer the conflict to the total ignorance assigned to the universal set of the frame of discernment denoted by m(Ω). Dubois and Prade [17] came up with a disjunctive combination rule that considers the union of the evidence rather than their intersection, and they assumed that for two sources, at least one of them is reliable. Smets [18] brought up a conjunctive combination rule also known as the un-normalized combination rule, where all the sources are considered reliable and the conflict is considered as a kind of information and it is allocated to the empty set m(ϕ). Lefevre et al. [19,20] used the part of the conflicting evidence and distributed the conflict into the focal element sets of all the evidence proportionally. These rules manage to solve the problem to some extent, but they lose both commutativity and associativity properties that are satisfied by the classical Dempster’s combination rule.

The second category believes that the problem of counter-intuitive results in conflict situations is caused by unreliable evidence rather than Dempster’s combination rule itself. A methodology aiming at pre-processing mass functions without changing the combination rule is then proposed. The main idea is to revise and reconstruct the evidence model in order to reduce the conflicting evidence impact on the final fusion result. To this end, diverse methods such as weighted averaging of mass functions are used. Weights are assigned to the original BPAs to determine their roles in the fusion process. On this basis, several weighted combination approaches have been proposed in the literature with various weight measurements [21,22,23,24,25,26,27,28,29,30,31,32,33,34,35,36,37,38,39,40,41,42,43,44,45,46,47,48,49,50,51,52,53,54,55,56,57,58,59,60,61,62,63,64,65,66,67,68,69,70,71,72,73,74,75,76,77,78,79,80,81,82,83,84,85,86,87,88,89,90,91]. The most well-known of them are summarized in Table 2 with their weights’ measurements.

## 4. Improved Evidence Distance

In this paper, we newly define an enhanced evidence distance based on Hellinger distance for D–S evidence theory by building on the concept of the belief functions transformation presented in [26]. The proposed evidence distance incorporates the correlation between bodies of evidence using a Jaccard matrix, thereby providing a more effective measure of the conflict degree between them. Furthermore, the improved evidence distance satisfies the requirements of a true metric, meeting non-negativity, positive definiteness, symmetry, and the triangle inequality properties.

It is worth noting that when all the elements are singletons, the mass function conforms to the classical probability distribution. In this case, the Jaccard matrix degenerates to the identity matrix, and, consequently, the improved evidence distance simplifies to the traditional Hellinger distance.

We define the improved evidence distance as follows:

(13) $$d_{IH}\left(m_{1},m_{2}\right)=\frac{1}{\sqrt{2}}\;\;\overset{n}{\underset{i=1}{\sum }}\|\sqrt{{m_{1}}^{\prime }\left(A_{i}\right)}-\sqrt{{m_{2}}^{\prime }\left(A_{i}\right)}{\|}_{2}$$

in which $m^{\prime }$ is expressed as follows:

(14) $$\left\{\begin{array}{c} m_{1}^{\prime }=m_{1}.D\\ m_{2}^{\prime }\;=m_{2}.D \end{array}\right.$$

where D is a Jaccard matrix of the size $2^{n}\times 2^{n}$, whose elements are obtained by the following:

(15) $$D\;\left(A_{i},A_{j}\right)=\frac{\left|A_{i}\cap A_{j}\right|}{\left|A_{i}\cup A_{j}\right|}\;\;\;\;A_{i},A_{j}\;\in 2^{\Omega }$$

The improved Hellinger distance satisfies the properties of non-negativity, symmetry, triangle inequality, and positive definiteness.

**Proof.**  In the following, the properties of non-negativity, symmetry, triangle inequality, and positive definiteness of the improved distance are verified.

The improved evidence distance Formula (13) can be written as follows:

(16) $$d_{IH}\left(m_{1},m_{2}\right)=\overset{n}{\underset{i=1}{\sum }}\sqrt{\frac{1}{2}{\left(\sqrt{{m_{1}}^{\prime }\left(A_{i}\right)}-\sqrt{{m_{2}}^{\prime }\left(A_{i}\right)}\right)}^{2}}$$

 □

**Proof.**  Non-negativity $0\leq \;d_{IH}\left(m_{1},m_{2}\right)\leq 1$:

$$\begin{array}{cc} d_{IH}^{2}\left(m_{1},m_{2}\right)= & \frac{1}{2}\;\sum_{i=1}^{n}\;{\left(\sqrt{m_{1}^{\prime }\left(A_{i}\right)}-\sqrt{m_{2}^{\prime }\left(A_{i}\right)\;}\;\right)}^{2}\\ & \;=\frac{1}{2}\;\sum_{i=1}^{n}\;\left(\sqrt{m_{1}^{\prime }\left(A_{i}\right)}-\sqrt{m_{2}^{\prime }\left(A_{i}\right)\;}\right)\left(\sqrt{m_{1}^{\prime }\left(A_{i}\right)}-\sqrt{m_{2}^{\prime }\left(A_{i}\right)\;}\right)\\ & \;\leq \;\frac{1}{2}\;\sum_{i=1}^{n}\;\left(\sqrt{m_{1}^{\prime }\left(A_{i}\right)}-\sqrt{m_{2}^{\prime }\left(A_{i}\right)\;}\right)\left(\sqrt{m_{1}^{\prime }\left(A_{i}\right)\;}+\sqrt{m_{2}^{\prime }\left(A_{i}\right)\;}\right)\\ & \;=\frac{1}{2}\;\sum_{i=1}^{n}\;\left({m_{1}}^{\prime }\left(A_{i}\right)-{m_{2}}^{\prime }\left(A_{i}\right)\right) \end{array}$$

Since *m* on Ω satisfies $0\leq m\left(A_{i}\right)\leq 1$, and the coefficient D ($A_{i},A_{j})$ of D has the property for all $A_{i},A_{j}$ of $2^{\Omega }$: 0≤ D ($A_{i},A_{j})\leq$1, we can deduce that $0\leq m^{\prime }\left(A_{i}\right)\leq 1$, thus

$$0\leq \sum_{i=1}^{n}\;\left({m_{1}}^{\prime }\left(A_{i}\right)-{m_{2}}^{\prime }\left(A_{i}\right)\right)\leq \;2$$

which implies the following:

$$0\leq \;d_{IH}\left(m_{1},m_{2}\right)\leq 1$$

The non-negativity property of the proposed evidence distance is proven. □

**Proof.**  Symmetry $d_{IH}\left(m_{1},m_{2}\right)=d_{IH}\left(m_{2},m_{1}\right)$. We have the following:

$$d_{IH}\left(m_{1},m_{2}\right)=\sum_{i=1}^{n}\;\sqrt{\frac{1}{2}{\left(\sqrt{m_{1}{}^{\prime }\left(A_{i}\right)}-\sqrt{m_{2}{}^{\prime }\left(A_{i}\right)\;}\;\right)}^{2}}$$

and,

$$d_{IH}\left(m_{2},m_{1}\right)=\sum_{i=1}^{n}\;\sqrt{\frac{1}{2}{\left(\sqrt{m_{2}{}^{\prime }\left(A_{i}\right)}-\sqrt{m_{1}{}^{\prime }\left(A_{i}\right)\;}\;\right)}^{2}}$$

It’s obvious that

$${\left(\sqrt{m_{2}{}^{\prime }\left(A_{i}\right)}-\sqrt{m_{1}{}^{\prime }\left(A_{i}\right)\;}\;\right)}^{2}={\left(\sqrt{m_{1}{}^{\prime }\left(A_{i}\right)}-\sqrt{m_{2}{}^{\prime }\left(A_{i}\right)\;}\;\right)}^{2}$$

thus,

$$\;\;\;\;\;\;\;\;d_{IH}\left(m_{1},m_{2}\right)=d_{IH}\left(m_{2},m_{1}\right)$$

therefore, the symmetry property of the proposed evidence distance is proven. □

**Proof.**  Triangle inequality:

$$d_{IH}\left(m_{1},m_{2}\right)+d_{IH}\left(m_{2},m_{3}\right)\;\geq \;d_{IH}\left(m_{1},m_{3}\right)\;d_{IH}\left(m_{1},m_{2}\right)+d_{IH}\left(m_{2},m_{3}\right)=\frac{1}{\sqrt{2}}{\left[\sum_{i=1}^{n}{\left(\sqrt{m_{1}{}^{\prime }\left(A_{i}\right)}-\sqrt{m_{2}{}^{\prime }\left(A_{i}\right)\;}\;\right)}^{2}\right]}^{\frac{1}{2}}+\frac{1}{\sqrt{2}}{\left[\sum_{i=1}^{n}{\left(\sqrt{m_{2}{}^{\prime }\left(A_{i}\right)}-\sqrt{m_{3}{}^{\prime }\left(A_{i}\right)\;}\;\right)}^{2}\right]}^{\frac{1}{2}}$$

We use Minkowski inequality [61]:

$${\left[\sum_{i=1}^{n}{\left(a_{i}+b_{i}\right)}^{p}\right]}^{\frac{1}{p}}\leq \;{\left[\sum_{i=1}^{n}{\left(a_{i}\right)}^{p}\right]}^{\frac{1}{p}}+{\left[\sum_{i=1}^{n}{\left(b_{i}\right)}^{p}\right]}^{\frac{1}{p}}\;$$

where p>1 and $a_{i},\;b_{i}>0$.

We receive the following:

$$d_{IH}\left(m_{1},m_{2}\right)+d_{IH}\left(m_{2},m_{3}\right)=\frac{1}{\sqrt{2}}{\left[\sum_{i=1}^{n}{\left(\sqrt{m_{1}{}^{\prime }\left(A_{i}\right)}-\sqrt{m_{2}{}^{\prime }\left(A_{i}\right)\;}\;\right)}^{2}\right]}^{\frac{1}{2}}+\frac{1}{\sqrt{2}}{\left[\sum_{i=1}^{n}{\left(\sqrt{m_{2}{}^{\prime }\left(A_{i}\right)}-\sqrt{m_{3}{}^{\prime }\left(A_{i}\right)\;}\;\right)}^{2}\right]}^{\frac{1}{2}}\;\;\;\;\;\;=\frac{1}{\sqrt{2}}\left\{{\left[\sum_{i=1}^{n}{\left(\sqrt{m_{1}{}^{\prime }\left(A_{i}\right)}-\sqrt{m_{2}{}^{\prime }\left(A_{i}\right)\;}\;\right)}^{2}\right]}^{\frac{1}{2}}+{\left[\sum_{i=1}^{n}{\left(\sqrt{m_{2}{}^{\prime }\left(A_{i}\right)}-\sqrt{m_{3}{}^{\prime }\left(A_{i}\right)\;}\;\right)}^{2}\right]}^{\frac{1}{2}}\right\}\;\geq \frac{1}{\sqrt{2}}\;{\left[\sum_{i=1}^{n}{\left(\sqrt{m_{1}{}^{\prime }\left(A_{i}\right)}-\sqrt{m_{2}{}^{\prime }\left(A_{i}\right)\;}\;\right)}^{2}+{\left(\sqrt{m_{2}{}^{\prime }\left(A_{i}\right)}-\sqrt{m_{3}{}^{\prime }\left(A_{i}\right)\;}\;\right)}^{2}\right]}^{\frac{1}{2}}$$

since:

$$\left(\sqrt{m_{1}{}^{\prime }\left(A_{i}\right)}-\sqrt{m_{2}{}^{\prime }\left(A_{i}\right)\;}\right)+\left(\sqrt{m_{2}{}^{\prime }\left(A_{i}\right)}-\sqrt{m_{3}{}^{\prime }\left(A_{i}\right)\;}\right)\geq \;\left(\sqrt{m_{1}{}^{\prime }\left(A_{i}\right)}-\sqrt{m_{3}{}^{\prime }\left(A_{i}\right)\;}\right)$$

we can deduce the following:

$$d_{IH}\left(m_{1},m_{2}\right)+d_{IH}\left(m_{2},m_{3}\right)\geq \frac{1}{\sqrt{2}}{\left[\sum_{i=1}^{n}{\left(\sqrt{m_{1}{}^{\prime }\left(A_{i}\right)}-\sqrt{m_{3}{}^{\prime }\left(A_{i}\right)\;}\;\right)}^{2}\right]}^{\frac{1}{2}}$$

and,

$$\frac{1}{\sqrt{2}}{\left[\sum_{i=1}^{n}{\left(\sqrt{m_{1}{}^{\prime }\left(A_{i}\right)}-\sqrt{m_{3}{}^{\prime }\left(A_{i}\right)\;}\;\right)}^{2}\right]}^{\frac{1}{2}}=d_{IH}\left(m_{1},m_{3}\right)\;$$

therefore,

$$d_{IH}\left(m_{1},m_{2}\right)+d_{IH}\left(m_{2},m_{3}\right)\geq d_{IH}\left(m_{1},m_{3}\right)\;$$

thus, the triangle inequality property of the proposed evidence distance is proven. □

**Proof.**  Positive definiteness $d_{IH}$($m_{1},m_{2}$) = 0, if and only if $m_{1}=m_{2}$:

$$\begin{array}{ccc} d_{IH}\left(m_{1},m_{2}\right)=0 & \Leftrightarrow & \frac{\|\sqrt{m_{1}{}^{\prime }\left(A_{i}\right)\;}-\sqrt{m_{2}{}^{\prime }\left(A_{i}\right)\;}{\|}_{2}}{\sqrt{2}}=0\\ & \Leftrightarrow & \|\sqrt{{m_{1}}^{\prime }\left(A_{i}\right)}-\sqrt{{m_{2}}^{\prime }\left(A_{i}\right)}{\|}_{2}=0\\ & \Leftrightarrow & {m_{1}}^{\prime }=\;{m_{2}}^{\prime }\\ & \Leftrightarrow & m_{1}.D=m_{2}.D\\ & \Leftrightarrow & m_{1}=m_{2} \end{array}$$

Finally, the positive definiteness property of the proposed evidence distance is proven. □

It can be seen from the above proofs that the improved evidence distance satisfies all the requirements, so it can be applied as a true metric under the Dempster–Shafer framework.

## 5. The Proposed Evidence Combination Method

To address the issue of the classical Dempster’s combination rule, we propose a new evidence combination method based on the revision of the evidence source model.

The proposed method utilizes (i) the newly defined improved evidence distance based on Hellinger distance to measure the conflict degree between the bodies of evidence and (ii) Deng entropy to quantify the uncertainty degree of each piece of evidence. These measurements are used to generate weighting factors that are applied to adjust the original pieces of evidence prior to the application of Dempster’s combination rule to get the final fusion results.

We evaluate the trustworthiness of the evidence based on a reliability condition set as the average credibility and design reward and penalty functions accordingly. We reward the reliable pieces of evidence by weighting them more heavily making their role more important in the final fusion result. On the other hand, we penalize unreliable pieces of evidence by using Deng entropy to diminish their impact on the final fusion result. The steps for our improved combination method are described as follows:

Step 1: According to Equation (13), the improved evidence distance between the two bodies of evidence $m_{i}$ (i = 1, 2, …, N) and $m_{j}$ (j = 1, 2, …, N) is calculated to measure the conflict degree.

The N × N distance matrix *D* is expressed below:

(17) $$D=\left[\begin{array}{cccc} 0 & d_{IH}\left(m_{1},m_{2}\right) & \ldots & d_{IH}\left(m_{1},m_{N}\right)\\ d_{IH}\left(m_{2},m_{1}\right) & 0 & \ldots & d_{IH}\left(m_{2},m_{N}\right)\\ \vdots & \vdots & \vdots & \vdots \\ d_{IH}\left(m_{N},m_{1}\right) & d_{IH}\left(m_{N},m_{2}\right) & \ldots & 0 \end{array}\right].\;$$

Step 2: Similarity degree between every two pieces of evidence is obtained using the formula proposed in [61] as follows:

(18) $$Sim\left(m_{i},m_{j}\right)=\left(1-\sqrt{d_{IH}\left(m_{i},m_{j}\right)}\right)e^{-\sqrt{d_{IH}\left(m_{i},m_{j}\right)}}$$

The N × N similarity matrix can now be written as follows:

(19) $$SIM=\left[\begin{array}{cccc} 1 & Sim\left(m_{1},m_{2}\right) & \ldots & Sim\left(m_{1},m_{N}\right)\\ Sim\left(m_{2},m_{1}\right) & 1 & \ldots & Sim\left(m_{2},m_{N}\right)\\ \vdots & \vdots & \vdots & \vdots \\ Sim\left(m_{N},m_{1}\right) & Sim\left(m_{N},m_{2}\right) & \ldots & 1 \end{array}\right]$$

Step 3: The support degree of each evidence can be evaluated using the previously calculated similarity degrees as follows:

(20) $$Sup\left(m_{i}\right)=\overset{N}{\underset{j=1,j\neq i}{\sum }}Sim(m_{i},\;m_{j})$$

Step 4: The degree of credibility reflects the level of trustworthiness associated with the evidence. The greater the credibility is, the more reliable the evidence; it is given by the following:

(21) $$CRD\left(m_{i}\right)=\frac{\;Sup(m_{i})}{\sum_{j=1}^{N}Sup\;(m_{j})}$$

Step 5: According to Equation (11), Deng entropy for each evidence is calculated to quantify the uncertainty degree.

Step 6: In this step, we establish a condition for determining reliability by setting a threshold that classifies pieces of evidence as either reliable or unreliable. The threshold rate is defined as follows:

(22) $$\alpha =\frac{\sum_{i=1}^{N}CRD\left(m_{i}\right)}{N}\;$$

When the credibility of a piece of evidence exceeds the threshold $\left(i.e.,\;CRD\left(m_{i}\right)\geq \;\alpha \right)$, the source is considered reliable. Conversely, if the credibility falls below the threshold $\left(i.e.,\;CRD\left(m_{i}\right)<\alpha \right)$, the source is deemed unreliable.

Step 7: Subsequently, we establish the initial weights by evaluating the fulfillment of the reliability condition. The objective is to increase the weights that surpass the threshold while decreasing the weights that fall below it, as outlined below:

If $CRD\left(m_{i}\right)\geq \;\alpha$*,* a reward function is defined as follows:

(23) $$w_{I}\left(m_{i}\right)=e^{E_{d}\left(m_{i}\right)}\;$$

If $CRD\left(m_{i}\right)<\alpha$*,* a penalty function is defined as follows:

(24) $$w_{I}\left(m_{i}\right)=e^{-\left(E_{dmax}+1-E_{d}\left(m_{i}\right)\right)}\;$$

Step 8: The final weights can be determined based on both credibility degree and the initial weight, resulting in the following definition:

(25) $$w\left(m_{i}\right)=\;\frac{CRD\left(m_{i}\right)\times w_{I}\left(m_{i}\right)}{\sum_{j=1}^{N}CRD\left(m_{j}\right)\times w_{I}\left(m_{j}\right)}\;$$

Thus, the weighted BPAs can be obtained by the following:

(26) $$m_{w}\left(A_{i}\right)=\sum_{j=1}^{N}w\left(m_{j}\right)\times m_{j}\left(A_{i}\right)\;$$

Step 9: Finally, for N body of evidence $m_{1},\;m_{2},\;\ldots ,m_{N}$, classical Dempster’s combination rule is applied N-1 times to get the final fusion result of the weighted BPAs.

(27) $$\left(m_{1}⊕\;m_{2}⊕\;\ldots ⊕m_{N}\right)\left(A_{i}\right)={\left({\left({\left({\left(m_{w}\left(A_{i}\right)\;⊕m_{w}\left(A_{i}\right)\right)}_{1}⊕\;m_{w}\left(A_{i}\right)\right)}_{2}⊕m_{w}\left(A_{i}\right)\right)}_{3}⊕\ldots ⊕m_{w}\left(A_{i}\right)\right)}_{\;N-1}$$

**Figure 1:**
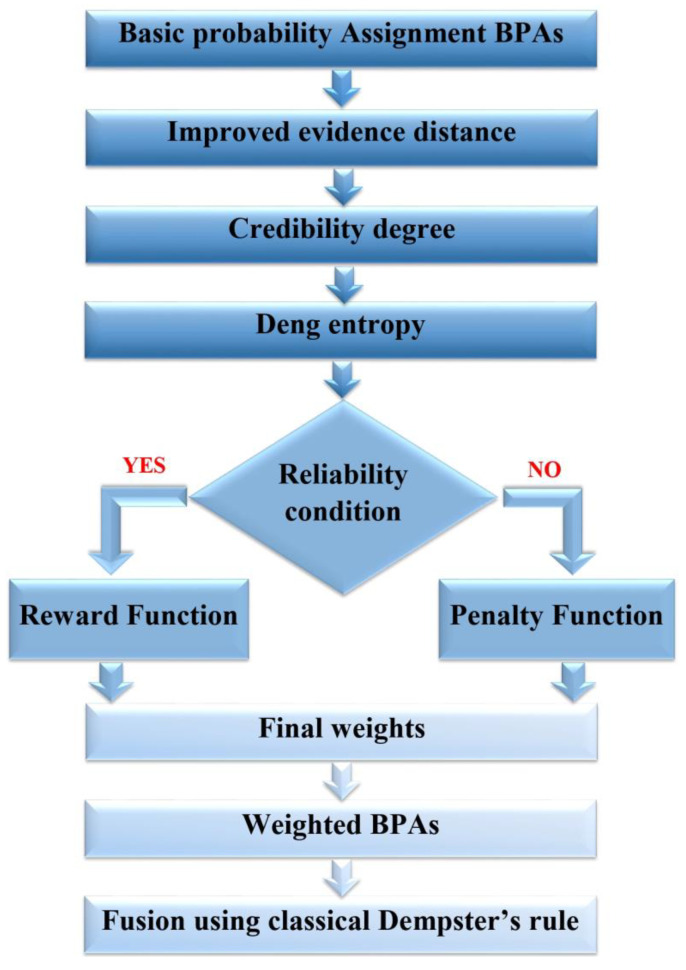
Flowchart of the proposed combination method.

The flowchart of the proposed approach is given in Figure 1.

## 6. Experiments and Analyses

In this section, we present a benchmark example for target recognition and two application cases for fault diagnosis and IoT decision-making to demonstrate the feasibility and effectiveness of our proposed method. Additionally, we have selected several similar methods from the existing literature to conduct a comparative analysis with the newly proposed approach.

### 6.1. Benchmark Example: Target Recognition

Similar to our prior research [86], we apply our newly proposed method to a benchmark example for target recognition. The fusion results are subsequently compared with those obtained from the Dempster–Shafer method (D–S) as well as several other similar methods selected from the literature, namely, methods from Murphy [21], Yong [22], Wang et al. [32], Yuan [34], and Yan et al. [68].

In a multisensor-based automatic target recognition system, based on five different kinds of sensors, CCD sensor (S_1_), sound sensor (S_2_), infrared sensor (S_3_), radar (S_4_), and ESM sensor (S5), three objects, A, B, and C, are detected. Let us suppose that the frame of discernment Ω = {A, B, C} is complete and A is the current target.

Sensor data modeled as BPAs are given in Table 3.

Based on the data presented in Table 3, it can be observed that S_2_ shows a strong conflict with other pieces of evidence, it assigns most of its belief to the wrong target B, while the remaining pieces of evidence mainly support the right target A. This situation may give rise to illogical results after combination using the classical Dempster’s rule, ultimately leading to the misidentification of the target.

**Figure 2:**
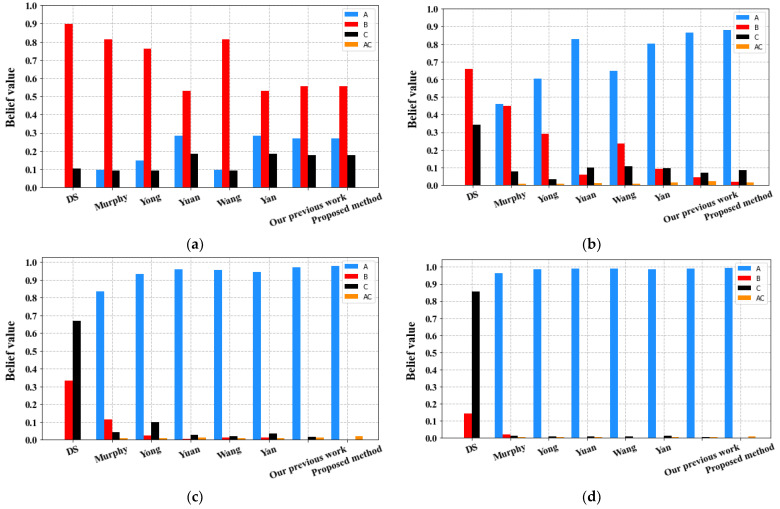
Fusion results in different methods for various numbers of evidence: (**a**) two pieces of evidence m_1_–m_2_; (**b**) three pieces of evidence m_1_–m_3_; (**c**) four pieces of evidence m_1_–m_4_; and (**d**) five pieces of evidence m_1_–m_5_.

Table 4 and Figure 2 depict the fusion results obtained from our proposed method and the other considered methods considered, using different numbers of evidence sources.

As observed from Table 3 and Figure 2, classical Dempster’s combination rule fails to identify the right target after combining data from the five sensors; results show that it assigns most of its belief wrongly to target C, while the belief assigned to A (the right target) remains at 0 regardless of the number of pieces of evidence considered. Obviously, the fusion process was disturbed by the abnormal source S_2_, highlighting the inadequacy of the classical Dempster’s combination rule in dealing with highly conflicting evidence.

Regarding the other fusion methods, including our proposed method, they also initially identify the wrong target B when only considering the two sensors S_1_ and S_2_, due to the conflicting evidence m_2_ that misguides the fusion process. However, as more sensors are included and more reliable pieces of evidence are considered (i.e., m_3_, m_4_, m_5_), all the methods achieve reasonable results and correctly identify the target A. 

**Figure 3:**
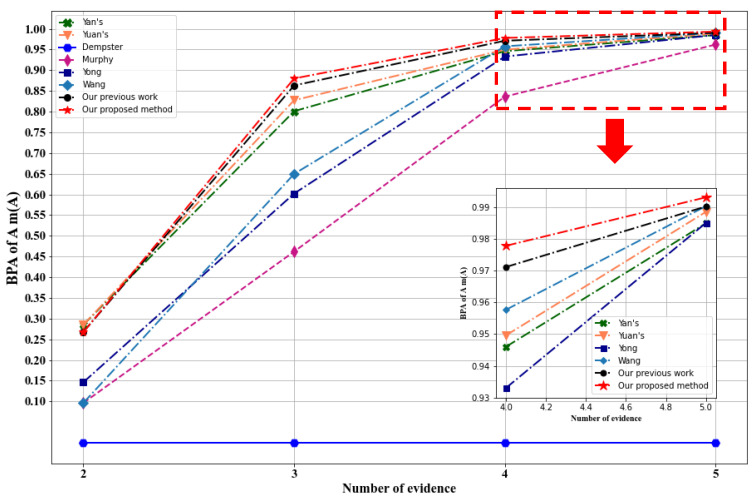
Comparison of the BPA of target A using different methods.

Figure 3 illustrates the evolution of the belief degree assigned to the right target A by the various methods after each combination of the five sensors. Although all methods eventually converge to A as the number of sensors increases, they do so at different rates (i.e., convergence speed) and with varying degrees of belief.

In summary, the proposed method exhibits superior performance compared to other methods, with the belief degrees of the right target A approaching 1 as the number of combined sensors increases. When five pieces of evidence are combined, it achieves the highest accuracy of 99.32% for identifying the right target, surpassing all other methods. It is worth noting that even a slight increase in accuracy is meaningful and represents a significant improvement in performance.

These results prove the effectiveness and superiority of our proposed method, it can handle the conflict between the pieces of evidence effectively, with the best convergence speed and decision accuracy. It is designed to evaluate the credibility of each piece of evidence, determine the importance of each sensor in the final fusion result, and assign the weights accordingly, which allows to overcome the influence of conflicting pieces of evidence, and therefore achieve better decision accuracy and fusion results reliability.

### 6.2. Application 1: Fault Diagnosis

To validate and demonstrate the effectiveness of the proposed method, a fault diagnosis application previously presented by Lin et al. [50] is used.

The fusion results of the proposed method are compared to those of the Dempster–Shafer method, our previous work [86], and other similar existing methods, including the Lin [50], Wang [54], and IDCR [73] methods.

In rotating machinery, the faults can be categorized into four types: F_1_ = “Imbalance”, F_2_ = “Shaft crack”, F_3_ = “Misalignment”, and F_4_ = “Bearing loose”. Therefore, the frame of discernment is Ω = {F_1_, F_2_, F_3_, F_4_}. To monitor the system status and determine the fault type, five different sensors were used.

Fault features were extracted from the data provided by the various sensors to calculate the BPAs of the five sensors. Table 5 illustrates the obtained results, which indicate that the fault diagnosis should be F3.

Data provided by S_1_, S_2_, and S_4_ indicate that the fault type is F_3_, whereas S_3_ and S_5_ indicate conflicting fault types, namely, F_2_ and F_4_, respectively.

**Figure 4:**
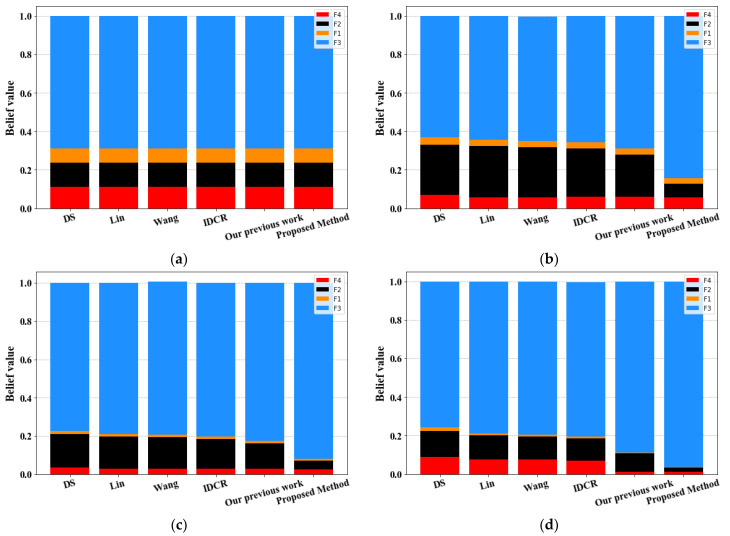
Fusion results in different methods for various numbers of evidence: (**a**) two pieces of evidence m_1_–m_2_; (**b**) three pieces of evidence m_1_–m_3_; (**c**) four pieces of evidence m_1_–m_4_; (**d**) five pieces of evidence m_1_–m_5_.

Fusion results obtained by varying the number of pieces of evidence for the different methods are shown in Table 6 and Figure 4.

**Figure 5:**
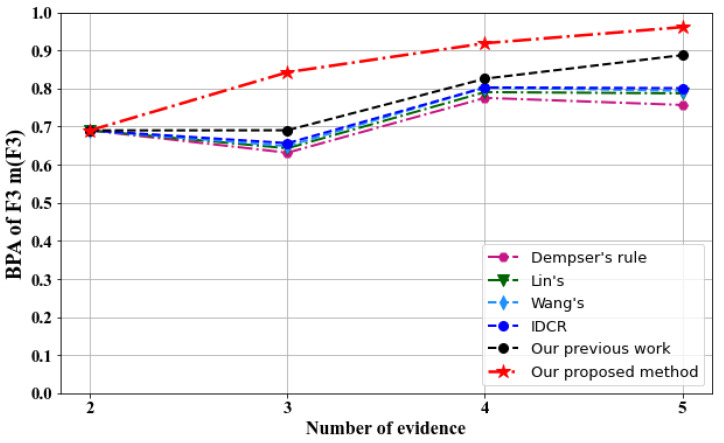
Comparison of the BPA of the fault F_3_ for the different methods used.

- Table 6 and Figure 4 show that all the methods, including our own, successfully diagnose the fault type as F_3_ after each combination of five pieces of evidence.
- Figure 5 depicts the evolution of the belief degree assigned to the right fault type F_3_ for the various compared methods using different numbers of sensors. Our proposed method exhibits superior performance compared to all other methods, as demonstrated in the figure.
- Combining three sensors results in a slight decrease in the belief degree of F_3_ for D–S, Lin [50], Wang [54], and IDCR [73] methods, which can be attributed to the conflicting data provided by S_3_. However, the inclusion of S_4_ in the fusion process causes the belief degree of F_3_ to increase again, reaching 0.7755 for D–S, 0.7906 for Lin, 0.8026 for Wang, and 0.8029 for IDCR, only to decrease once more when S_5_ is incorporated in the fusion process for all methods.
- However, both the method proposed in our previous work [86] and the newly proposed approach maintain the accurate fusion performance, as the belief degree of the right fault type F_3_ continues to increase despite the inclusion of the conflicting sources S_3_ and S_5_.
- Upon combining five sensors, our proposed method achieves the highest belief degree for F_3_ with a value of 0.9614, which is greater than the maximum value achieved by any other method, including our previous work, which did not exceed 0.90.

### 6.3. Application 2: IoT Decision-Making

An IoT decision-making application from the work of Boulkaboul et al. [64] is considered. The model was evaluated through a set of experiments applied in IoT and smart building projects, realized in the CERIST-ALGERIA research center laboratory.

In the considered scenario, IoT-enabled wireless sensors are used to monitor office occupancy and ambient light to control electrical lighting and optimize energy, and data fusion method is applied to make a decision about the light switch ON/OFF.

Three (03) PIR (Passive Infrared) sensors S_1_, S_2_, S_3_, and a light sensor S_4_ have been placed in optimal positions on the ceiling of the office and four hypotheses (H_1_, H_2_, H_3_, H_4_) are defined as follows: H_1_: the office is occupied, and the lighting value exceeds 580 lx; H_2_: the office is empty, and the lighting value exceeds 580 lx; H_3_: the office is occupied, and the lighting value does not exceed 580 lx; and H_4_: the office is empty, and the lighting value does not exceed 580 lx. A basic scenario is considered when hypothesis H_1_ is verified, and the system has generated evidence when hypothesis H_1_ occurs.

In [64], the impact of the environment on the generation of evidence has not been considered. Therefore, in [94], 10% of the belief degree was assigned to Ω to represent a completely unknown situation. The BPAs obtained from data generated by the four sensors are depicted in Table 7, where the frame of discernment is defined as follows: Ω = {H_1_, H_2_, H_3_, H_4_}.

**Figure 6:**
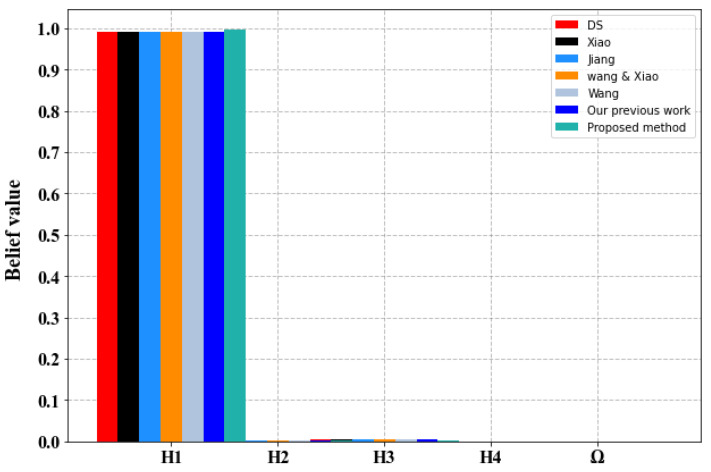
Comparison of fusion results using four sensors in different methods.

We use the proposed method to combine the pieces of evidence and we compare the results obtained with Dempster–Shafer method (D–S) and six of similar existing methods including the following: Wang [94], Xiao [95], Jiang et al. [96], Wang and Xiao [97] methods, and our previous work [86]. Fusion results are depicted in Table 8 and Figure 6.

**Figure 7:**
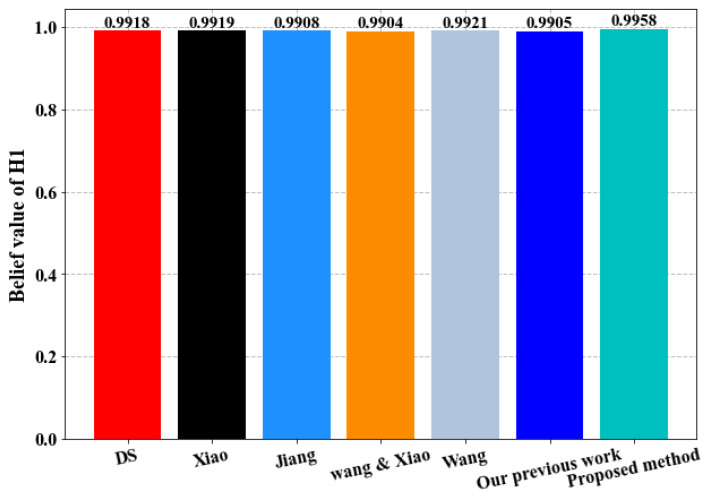
Comparison of the belief value for the hypothesis H_1_ in different methods.

- As evident from Figure 6 and Table 8, all the techniques, including the Dempster–Shafer method, are capable of identifying the correct hypothesis H_1_ due to the absence of substantial conflicts among the pieces of evidence.
- Prior to the combination of the pieces of evidence, none of the sensors in Table 6 report a belief degree of more than 0.75 for the correct hypothesis H_1_. However, upon employing various combination methods, including the proposed approach, it is evident that the results converge to yield high belief degrees.
- It is worth noting that our proposed combination method delivers significantly better results, providing stronger support for the right hypothesis H_1_ than the other methods. This is evidenced by the maximum belief degree of 0.9958, as illustrated in Figure 7
- The primary reason for this enhancement is that our proposed method accounts for the relevance and disparities among the pieces of evidence, which is enabled by the improved evidence distance. By identifying even minor conflicts among the pieces of evidence and assigning appropriate weights accordingly, the influence of untrustworthy pieces of evidence is reduced, and that of reliable evidence is increased. This leads to a significant improvement in convergence and decision accuracy.

## 7. Conclusions

In this paper, an improved evidence combination method for multisensor data fusion in IoT environments has been proposed that aims to address the conflict issues encountered in the Dempster–Shafer theory. The approach relies on the revision of the evidence source model; it is devised to greatly reduce the conflicting impact of unreliable pieces of evidence while strengthening the influence of reliable ones on the final fusion results. To this end, an improved evidence distance based on the Hellinger distance was introduced to measure the degree of conflict among the pieces of evidence, while Deng entropy was employed to quantify the degree of uncertainty of each piece of evidence. The original mass functions are then adjusted using comprehensive weights derived from these metrics, and the classical Dempster’s combination rule is then applied to obtain the final fusion results.

To demonstrate the effectiveness of the proposed method, a benchmark example for target recognition and two real application cases in Fault diagnosis and IoT decision-making have been provided. Several comparable methods have been selected from the literature to conduct a comparative analysis with the newly proposed approach, and simulation analyses have shown the efficiency and superiority of the proposed method in terms of conflict management, convergence speed, fusion results reliability, and decision accuracy. In fact, the proposed approach effectively achieved the highest accuracy rate of 99.32% in correctly identifying the target compared to other considered methods in the benchmark example. It also demonstrated a better performance in converging to the right fault type, with a remarkable accuracy rate of 96.14% in the fault diagnosis problem. Furthermore, in the IoT decision-making application, it correctly recognized the right hypothesis and outperformed other related methods, achieving the highest accuracy rate of 99.58%

For future research directions, we intend to apply the proposed method on larger data to demonstrate its broader applicability; we also plan to further improve the proposed method by considering more relevant factors in weighting factors’ construction such as data timeliness. The proposed method is only applicable in the closed-world situation, so adjusting the approach in order to obtain reasonable and accurate results in an open-world assumption is also a promising axis.

## Figures and Tables

**Table 1:** List of symbols and abbreviations.

Abbreviation	Semantic	Abbreviation	Semantic
D–S	Dempster–Shafer theory	$E_{d}$	Deng etropy
Ω	Frame of discernment	$\left|A_{i}\right|$	Cardinality of a subset
2^Ω^	Power set	D	Jaccard matrix
m	Mass function	Sim	Similarity degree
BPA	Basic Probability Assignment	Sup	Support degree
Bel	Belief function	CRD	Credibility degree
Pl	Plausibility function	α	Threshold rate
K	Conflict coefficient	N	Number of pieces of evidence
$d_{H}$	Hellinger distance	n	Number of hypotheses
$d_{IH}$	Improved Evidence Distance	$w_{I}$	Initial weights
$d_{J}$	Jousselme distance	w	Final weights

**Table 2:** Improved weighted average approaches.

Weighted Approaches	Weights’ Measurements	
	Conflict Measure	Uncertainty Measure
Yong et al. [22]	Jousselme distance $d_{J}\left(m_{i},m_{j}\right)=\sqrt{\;\frac{1}{2}{\left(\vec{m_{i}}-\vec{m_{j}}\right)}^{T}\;D\left(\vec{m_{i}}-\vec{m_{j}}\right)}$	/
Zhang [25]	Cosine value of pignistic vectors $Cos\theta =\frac{Pignistic\;vector\;m_{i},\;Pignistic\;vector\;m_{j}}{\left|Pignistic\;vector\;m_{i}\right|\times \left|Pignistic\;vector\;m_{j}\right|}$	/
Yu et al. [29]	New supporting probability function of evidence $SPFE=\underset{A,B\subseteq \Omega }{\sum }\frac{\left|A\cap B\right|}{\left|A\right|}m\left(B\right)$	/
Jing [30]	Generalized Mahalanobis distance ${\mathrm{GD}}_{M}\left(m_{i},m_{j}\right)=\sqrt{\;\frac{1}{2}{\left(m_{i}-m_{j}\right)}^{T}P^{+}\left(m_{i}-m_{j}\right)}$	/
Li et al. [31]	Minkowski distance $d_{J}\left(m_{i},m_{j}\right)={\left(\overset{n}{\underset{1}{\sum }}{\left|m_{i}-m_{j}\right|}^{m}\right)}^{1/m}$	/
Yuan et al. [34]	Jousselme distance $d_{J}\left(m_{i},m_{j}\right)=\sqrt{\;\frac{1}{2}{\left(\vec{m_{i}}-\vec{m_{j}}\right)}^{T}\;D\left(\vec{m_{i}}-\vec{m_{j}}\right)}$	Deng entropy $E_{d}=-\sum_{i}m\left(A_{i}\right)log\frac{m\left(A_{i}\right)}{2^{\left|A_{i}\right|}-1\;}\;\;$
Ye et al. [36]	1. Lance distance function: $d_{ij}\left(L\right)=\left(\frac{1}{n}\overset{n}{\underset{1}{\sum }}\frac{\left|m_{i}-m_{j}\right|}{\left(m_{i}+m_{j}\right)}\right)$ 2. Spectral angle cosine function: $\cos\left(m_{i}.m_{j}\right)=\frac{\langle \vec{m_{i}},\vec{m_{j}}\rangle }{\left\|\vec{m_{i}}\|.\|\vec{m_{j}}\right\|}$	/
Tang et al. [37]	/	Weighted belief entropy $E_{Id}=-\sum_{A\subseteq \Omega }m\left(A_{i}\right)log\frac{m\left(A_{i}\right)}{2^{\left|A_{i}\right|}-1\;}e^{\frac{\left|A_{i}\right|-1}{\left|\Omega \right|}}\;$
Lin et al. [50]	Euclidean distance $d\left(m_{I},m_{avg}\right)=\sqrt{\;\overset{n}{\underset{j=1}{\sum }}{\left[m_{i}\left(A_{j}\right)-m_{\mathrm{avg}}\left(A_{j}\right)\right]}^{2}}$	/
Li et al. [61]	Hellinger distance $d_{H}\left(m_{i},m_{j}\right)=\frac{1}{\sqrt{2}}\overset{n}{\underset{i=1}{\sum }}\|\sqrt{m_{1}\left(A_{i}\right)}-\sqrt{m_{2}\left(A_{i}\right)}{\|}_{2}$	New belief entropy $\;E_{x}=-\sum_{A\subseteq \Omega }m\left(A_{i}\right)log\frac{m\left(A_{i}\right)}{2^{\left|A_{i}\right|}-1\;}e^{\frac{\left|A_{i}\right|-1}{2^{\left|X\right|}-1}}\;\;$
Deng et al. [63]	1. Hellinger distance 2. Sine value of the pignistic vectors $Sin\;\left(m_{i},m_{j}\right)=\sqrt{1-{\left(\frac{BetPm_{i}.BetPm_{j}}{\left\|BetPm_{i}\|.\|BetPm_{j}\right\|}\right)}^{2}}$	/
Boulkaboul et al. [64]	Jousselme distance $d_{J}\left(m_{i},m_{j}\right)=\sqrt{\;\frac{1}{2}{\left(\vec{m_{i}}-\vec{m_{j}}\right)}^{T}\;D\left(\vec{m_{i}}-\vec{m_{j}}\right)}$	Improved Deng entropy $\;E_{d}=1-\frac{\;\;\;E_{d}}{sum\left(\;E_{d}\right)}$
Li et al. [65]	Improved Jousselme distance $d_{i}\left(m_{i},m_{j}\right)=\sqrt{\;\frac{1}{2}{\left(\vec{m_{i}}-\vec{m_{j}}\right)}^{T}D_{i}\;\left(\vec{m_{i}}-\vec{m_{j}}\right)}\;D_{i}\left(S_{1},S_{2}\right)=\frac{2^{\left|S_{1}\cap S_{2}\right|}-1}{\sqrt{\left(2^{\left|S_{1}\right|}-1)(2^{\left|S_{2}\right|}-1\right)}}$	Tssalis entropy $T_{DS}=\underset{A_{i}\subseteq 2^{\Omega }}{\sum }\frac{\left(2^{\left|A_{i}\right|}-1\right)\times m\left(A_{i}\right)\times {\left(\frac{m\left(A_{i}\right)}{2^{\left|A_{i}\right|}-1\;}\right)}^{\left|A_{i}\right|-1}}{\left|A_{i}\right|-1}$
Sun et al. [66]	Pignistic probability distance $DifBet\left(m_{i},m_{j}\right)=\underset{A_{i}\subseteq \Omega }{\max}\left(\left|Bet_{m_{i}}\left(A_{i}\right)-Bet_{m_{j}}\left(A_{i}\right)\right|\right)$	Deng entropy $E_{d}=-\sum_{i}m\left(A_{i}\right)log\frac{m\left(A_{i}\right)}{2^{\left|A_{i}\right|}-1\;}\;$
Yan et al. [68]	/	New belief entropy $H_{n}=-\sum_{A\subseteq \Omega }m\left(A_{i}\right)log\frac{m\left(A_{i}\right)+bel\left(A_{i}\right)}{2\left(2^{\left|A_{i}\right|}-1\right)}e^{\frac{\left|A_{i}\right|-1}{\left|C\right|}}\;$
Chen et al. [71]	1. Jousselme distance 2. Evidence angle $Cos\;\left(m_{i},m_{j}\right)=\frac{BetPm_{i}.BetPm_{j}}{\left\|BetPm_{i}\|.\|BetPm_{j}\right\|}$	Improved entropy function $\;E_{i}=-\sum_{i}m\left(A_{i}\right)log\frac{m\left(A_{i}\right)}{2^{\left|A_{i}\right|}-1\;}\frac{\left|A_{i}\right|}{\left|C\right|}\;\;$
Ghosh et al. [73]	Euclidean distance $d\left(m_{i},m_{\mathrm{avg}}\right)=\sqrt{\;\overset{n}{\underset{j=1}{\sum }}{\left[m_{i}\left(A_{j}\right)-m_{\mathrm{avg}}\left(A_{j}\right)\right]}^{2}}$	Weighted Deng entropy $\;E_{wd}=-\sum_{i}\frac{\left|A_{i}\right|.m\left(A_{i}\right)}{\left|\Omega \right|}log\frac{m\left(A_{i}\right)}{2^{\left|A_{i}\right|}-1\;}\;$
Xiao et al. [75]	Evidential correlation coefficient (ECC) $ECC={\left[\frac{\langle \vec{m_{i}},\vec{m_{j}}\rangle }{\left\|\vec{m_{i}}\|.\|\vec{m_{j}}\right\|}\right]}^{2}$	/
Zhu et al. [78]	Belief Hellinger distance $BH\left(m_{1},m_{2}\right)=\frac{1}{\sqrt{2}}\overset{n}{\underset{i=1}{\sum }}\frac{\sqrt{m_{1}\left(A_{i}\right)}-\sqrt{m_{2}\left(A_{i}\right)}}{2^{\left|A_{i}\right|}-1}$	Deng entropy $\;E_{d}=-\sum_{i}m\left(A_{i}\right)log\frac{m\left(A_{i}\right)}{2^{\left|A_{i}\right|}-1\;}\;\;$
Ullah et al. [79]	/	Modified belief entropy $E_{p}\left(m\right)=-\sum_{i}m\left(A^{\prime }\right)log\frac{m\left(A^{\prime }\right)}{2^{\left|A^{\prime }\right|}-1\;}\;\;\;\;A^{\prime }=m\left(A_{i}\right)\;\cup \;m(A_{j})$
Zhao [80]	Distribution based on the Squared mean of entropy $DSM\left(m_{1},m_{2}\right)=\sqrt{\frac{{\left[\sum_{i}m_{1}\left(A_{i}\right)\log\sqrt{\frac{2m_{1}{\left(A_{i}\right)}^{2}}{m_{1}{\left(A_{i}\right)}^{2}+m_{2}{\left(A_{i}\right)}^{2}}}\;\;\;\;\right]}^{2}+{\left[\sum_{i}m_{2}\left(A_{i}\right)\log\sqrt{\frac{2m_{2}{\left(A_{i}\right)}^{2}}{m_{1}{\left(A_{i}\right)}^{2}+m_{2}{\left(A_{i}\right)}^{2}}}\;\;\;\;\right]}^{2}}{2}}$	Modified Entropy $E=-\sum_{A\subseteq \Omega }m\left(A_{i}\right)log\frac{m\left(A_{i}\right)}{2^{\left|A_{i}\right|}-1}e^{\underset{\begin{array}{c} A_{j}\subseteq \Omega \\ A_{j}\neq A_{i} \end{array}}{\sum }\frac{\left|A_{i}\cap A_{j}\right|}{2^{\left|A_{i}\right|}}}\;\;\;\;$
Hamda et al. [86]	1. Jousselme distance 2. Evidence angle $Cos\left(\vec{m_{i}},\vec{m_{j}}\right)=\frac{\langle \vec{m_{i}},\vec{m_{j}}\rangle }{\left\|\vec{m_{i}}\|.\|\vec{m_{j}}\right\|}$	Deng entropy $E_{d}=-\sum_{i}m\left(A_{i}\right)log\frac{m\left(A_{i}\right)}{2^{\left|A_{i}\right|}-1\;}\;\;\;$

**Table 3:** BPAs of the benchmark example (Where AC stands for {A, C}).

	A	B	C	AC
S_1_:m_1_ (.)	0.41	0.29	0.3	0
S_2_:m_2_ (.)	0	0.9	0.1	0
S_3_:m_3_ (.)	0.58	0.07	0	0.35
S_4_:m_4_ (.)	0.55	0.1	0	0.35
S_5_:m_5_ (.)	0.6	0.1	0	0.3

**Table 4:** Fusion results by different methods for various numbers of evidence.

Methods	Fusion Results				Target
	m_1_–m_2_	m_1_–m_3_	m_1_–m_4_	m_1_–m_5_	
D–S	m(A) = 0 m(B) = 0.8969 m(C) = 0.1031	m(A) = 0 m(B) = 0.6575 m(C) = 0.3425	m(A) = 0 m(B) = 0.3321 m(C) = 0.6679	m(A) = 0 m(B) = 0.1422 m(C) = 0.8578	C
Murphy [21]	m(A) = 0.0964 m(B) = 0.8119 m(C) = 0.0917 m(AC) = 0	m(A) = 0.4619 m(B) = 0.4497 m(C) = 0.0794 m(AC) = 0.0090	m(A) = 0.8362 m(B) = 0.1147 m(C) = 0.0410 m(AC) = 0.0081	m(A) = 0.9620 m(B) = 0.0210 m(C) = 0.0138 m(AC) = 0.0032	A
Yong [22]	m(A) = 0.1463 m(B) = 0.7620 m(C) = 0.0917 m(AC) = 0	m(A) = 0.6021 m(B) = 0.2907 m(C) = 0.0353 m(AC) = 0.0082	m(A) = 0.9330 m(B) = 0.0225 m(C) = 0.0990 m(AC) = 0.0092	m(A) = 0.9851 m(B) = 0.0017 m(C) = 0.0096 m(AC) = 0.0035	A
Yuan [34]	m(A) = 0.2849 m(B) = 0.5306 m(C) = 0.1845 m(AC) = 0	m(A) = 0.8274 m(B) = 0.0609 m(C) = 0.0986 m(AC) = 0.0131	m(A) = 0.9596 m(B) = 0.0032 m(C) = 0.0267 m(AC) = 0.0106	m(A) = 0.9886 m(B) = 0.0002 m(C) = 0.0072 m(AC) = 0.0039	A
Wang et al. [32]	m(A) = 0.0964 m(B) = 0.8119 m(C) = 0.0917 m(AC) = 0	m(A) = 0.6495 m(B) = 0.2367 m(C) = 0.1065 m(AC) = 0.0079	m(A) = 0.9577 m(B) = 0.0129 m(C) = 0.0200 m(AC) = 0.0094	m(A) = 0.9904 m(B) = 0.0009 m(C) = 0.0068 m(AC) = 0.0019	A
Yan et al. [68]	m(A) = 0.2850 m(B) = 0.5310 m(C) = 0.1840 m(AC) = 0	m(A) = 0.8010 m(B) = 0.0910 m(C) = 0.0950 m(AC) = 0.0140	m(A) = 0.9460 m(B) = 0.0110 m(C) = 0.0340 m(AC) = 0.0090	m(A) = 0.9850 m(B) = 0.0010 m(C) = 0.0110 m(AC) = 0.0030	A
Our previous work [86]	m(A) = 0.2678 m(B) = 0.5551 m(C) = 0.1771 m(AC) = 0	m(A) = 0.8632 m(B) = 0.0449 m(C) = 0.0687 m(AC) = 0.0231	m(A) = 0.9712 m(B) = 0.0010 m(C) = 0.0142 m(AC) = 0.0136	m(A) = 0.9903 m(B) = 0.0001 m(C) = 0.0049 m(AC) = 0.0047	A
Our proposed method	m(A) = 0.2677 m(B) = 0.5552 m(C) = 0.1770 m(AC) = 0	m(A) = 0.8800 m(B) = 0.0179 m(C) = 0.0859 m(AC) = 0.0161	m(A) = 0.9780 m(B) = 0.00009 m(C) = 0.0011 m(AC) = 0.0208	m(A) = 0.9932 m(B) = 0.00001 m(C) = 0.0002 m(AC) = 0.0065	A

**Table 5:** BPAs modeled from the five sensors.

	F_1_	F_2_	F_3_	F_4_
S_1_:m_1_ (.)	0.1469	0.2057	0.4660	0.1813
S_2_:m_2_ (.)	0.1521	0.1935	0.4631	0.1914
S_3_:m_3_ (.)	0.1278	0.5008	0.2221	0.1493
S_4_:m_4_ (.)	0.1459	0.2396	0.4395	0.1750
S_5_:m_5_ (.)	0.2068	0.1399	0.1755	0.4777

**Table 6:** Comparison of the fusion results based on different combination rules.

Methods	Fusion Results			
	m_1_–m_2_	m_1_–m_3_	m_1_–m_4_	m_1_–m_5_
D–S	m($F_{1}$) = 0.0714 m($F_{2}$) = 0.1273 m($F_{3}$) = 0.6902 m($F_{4}$) = 0.1110	m($F_{1}$) = 0.0376 m($F_{2}$) = 0.2626 m($F_{3}$) = 0.6315 m($F_{4}$) = 0.0683	m($F_{1}$) = 0.0153 m($F_{2}$) = 0.1758 m($F_{3}$) = 0.7755 m($F_{4}$) = 0.0334	m($F_{1}$) = 0.0176 m($F_{2}$) = 0.1368 m($F_{3}$) = 0.7570 m($F_{4}$) = 0.0886
Lin et al. [50]	m($F_{1}$) = 0.0715 m($F_{2}$) = 0.1274 m($F_{3}$) = 0.6903 m($F_{4}$) = 0.1111	m($F_{1}$) = 0.0315 m($F_{2}$) = 0.2675 m($F_{3}$) = 0.6431 m($F_{4}$) = 0.0579	m($F_{1}$) = 0.0125 m($F_{2}$) = 0.1692 m($F_{3}$) = 0.7906 m($F_{4}$) = 0.0276	m($F_{1}$) = 0.0109 m($F_{2}$) = 0.1258 m($F_{3}$) = 0.7874 m($F_{4}$) = 0.0759
Wang et al. [54]	m($F_{1}$) = 0.0715 m($F_{2}$) = 0.1274 m($F_{3}$) = 0.6900 m($F_{4}$) = 0.1110	m($F_{1}$) = 0.0314 m($F_{2}$) = 0.2594 m($F_{3}$) = 0.6490 m($F_{4}$) = 0.0578	m($F_{1}$) = 0.0126 m($F_{2}$) = 0.1643 m($F_{3}$) = 0.8026 m($F_{4}$) = 0.0278	m($F_{1}$) = 0.0108 m($F_{2}$) = 0.1204 m($F_{3}$) = 0.7941 m($F_{4}$) = 0.0747
IDCR [73]	m($F_{1}$) = 0.0715 m($F_{2}$) = 0.1274 m($F_{3}$) = 0.6901 m($F_{4}$) = 0.1111	m($F_{1}$) = 0.0315 m($F_{2}$) = 0.2540 m($F_{3}$) = 0.6565 m($F_{4}$) = 0.0585	m($F_{1}$) = 0.0124 m($F_{2}$) = 0.1571 m($F_{3}$) = 0.8029 m($F_{4}$) = 0.0275	m($F_{1}$) = 0.0103 m($F_{2}$) = 0.1148 m($F_{3}$) = 0.8011 m($F_{4}$) = 0.0692
Our previous work [86]	m($F_{1}$) = 0.0715 m($F_{2}$) = 0.1273 m($F_{3}$) = 0.6901 m($F_{4}$) = 0.1111	m($F_{1}$) = 0.0314 m($F_{2}$) = 0.2198 m($F_{3}$) = 0.6905 m($F_{4}$) = 0.0583	m($F_{1}$) = 0.0122 m($F_{2}$) = 0.1348 m($F_{3}$) = 0.8259 m($F_{4}$) = 0.0271	m($F_{1}$) = 0.0046 m($F_{2}$) = 0.0950 m($F_{3}$) = 0.8878 m($F_{4}$) = 0.0125
Our proposed method	m($F_{1}$) = 0.0715 m($F_{2}$) = 0.1274 m($F_{3}$) = 0.6901 m($F_{4}$) = 0.1110	m($F_{1}$) = 0.0285 m($F_{2}$) = 0.0733 m($F_{3}$) = 0.8429 m($F_{4}$) = 0.0552	m($F_{1}$) = 0.0104 m($F_{2}$) = 0.0467 m($F_{3}$) = 0.9190 m($F_{4}$) = 0.0238	m($F_{1}$) = 0.0037 m($F_{2}$) = 0.0237 m($F_{3}$) = 0.9614 m($F_{4}$) = 0.0111

**Table 7:** BPAs modeled from the four sensors.

	H_1_	H_2_	H_3_	H_4_	Ω
S_1_:m_1_ (.)	0.729	0.054	0.099	0.018	0.1
S_2_:m_2_ (.)	0.747	0.063	0.081	0.009	0.1
S_3_:m_3_ (.)	0.648	0.153	0.09	0.009	0.1
S_4_:m_4_ (.)	0.621	0.072	0.198	0.009	0.1

**Table 8:** Fusion results using different methods.

Methods	Fusion Results				
	H_1_	H_2_	H_3_	H_4_	Ω
D–S	0.9918	0.0027	0.0051	0.0001	0.0003
Wang et al. [94]	0.9921	0.0025	0.0050	0.0001	0.0003
Xiao [95]	0.9919	0.0026	0.0051	0.0001	0.0003
Jiang et al. [96]	0.9908	0.0030	0.0058	0.0001	0.0003
Wang and Xiao [97]	0.9904	0.0031	0.0061	0.0001	0.0003
Our previous work [86]	0.9905	0.003	0.0060	0.0001	0.0003
Our proposed method	0.9958	0.0012	0.0027	0.0001	0.0002

## Data Availability

All relevant data are within the paper.
